# Supplementary material for: A high-resolution mRNA expression time course of embryonic development in zebrafish
Source: eLife. 2017 Nov 16;6:e30860. doi: 10.7554/eLife.30860 (PMC5690287; doi:10.7554/eLife.30860)
Supplement: Supplementary file 6. [file elife-30860-supp6.zip › biolayout-clusters-files/Cluster172-genes.html]

Cluster172


# Cluster172: Genes

| Ensembl ID | Gene Name | Chr | Start | End | Biotype |
| --- | --- | --- | --- | --- | --- |
| ENSDARG00000061168 | dhx34 | 18 | 44573737 | 44596638 | protein\_coding |
| ENSDARG00000039345 | drg1 | 10 | 17723640 | 17733737 | protein\_coding |
| ENSDARG00000067979 | gtf3c5 | 21 | 11274625 | 11290480 | protein\_coding |
| ENSDARG00000016753 | ipo9 | 11 | 24614055 | 24639005 | protein\_coding |
| ENSDARG00000056318 | kansl2 | 23 | 24672369 | 24682953 | protein\_coding |
| ENSDARG00000104771 | timm44 | 22 | 3994583 | 4010640 | protein\_coding |
| ENSDARG00000027141 | ube2l3b | 5 | 12837290 | 12853688 | protein\_coding |
| ENSDARG00000012215 | umps | 9 | 11605299 | 11615867 | protein\_coding |
